# Comparing the Effects of Baclofen, Transcutaneous Electrical Nerve Stimulation, and Sustained Stretch for Treating Spasticity After Traumatic Spinal Cord Injury: A Randomized Clinical Trial

**DOI:** 10.7759/cureus.100840

**Published:** 2026-01-05

**Authors:** Bazmeer Afridi, Saima Gul, Zoya Mahmood, Muhammad Suleman Sikander, Khalil ur Rehman, Fouzia Batool

**Affiliations:** 1 Department of Physical Therapy, City University of Science and Information Technology, Peshawar, PAK; 2 Department of Rehabilitation Sciences, Shifa Tameer-e-Millat University, Islamabad, PAK; 3 Department of Physiotherapy, Hayatabad Medical Complex Peshawar, Peshawar, PAK

**Keywords:** baclofen, rehabilitation, spasticity, spinal cord injury, sustained stretch, tens

## Abstract

Objective

The main objective of this study is to evaluate and compare the effectiveness of baclofen, transcutaneous electrical nerve stimulation (TENS), and sustained stretch in managing spasticity in traumatic spinal cord injury (SCI) patients.

Materials and methods

A single-blinded randomized clinical trial was conducted for a duration of six months at the Paraplegic Center, Peshawar, with IRB approval (no. 082-21) from Shifa Tameer-e-Millat University (STMU), Islamabad, Pakistan. Patients were randomly assigned to one of three intervention groups: baclofen group (45 mg/day), TENS group (20 min/day), and sustained stretch group (20 sec × 20 reps/day) targeting the gastrocnemius muscle. Data were collected at baseline and at the first, second, third, and fourth weeks post-intervention.

Data were analyzed using IBM SPSS Statistics for Windows, Version 24 (Released 2016; IBM Corp., Armonk, NY, USA). Normality was assessed via the Shapiro-Wilk test. Friedman’s two-way analysis of variance by rank was used to evaluate within-group differences over time, while the Kruskal-Wallis test compared the three interventions at different time points. Post hoc Bonferroni correction analysis was applied to confirm the significance level.

Results

A total of 63 patients (44 males (69.84%) and 19 females (30.16%)) with a mean age of 30.75 ± 9.02 years were included. Significant improvements (p < 0.001, with a 95% confidence interval) in spasticity between baseline and the first, second, third, and fourth weeks of intervention were observed in all intervention groups. TENS demonstrated a significant difference between baseline and the fourth week (p < 0.05), but no significant differences between intermediate weeks (p > 0.05).

Conclusion

All interventions effectively reduced spasticity. Baclofen provided the fastest relief, while sustained stretch showed delayed but comparable efficacy, without adverse effects. These findings suggest that sustained stretch may serve as a non-pharmacological alternative to baclofen for managing spasticity in SCI patients.

## Introduction

Spinal cord injury (SCI), caused by trauma, disease, or degeneration, significantly impacts all aspects of a person's life, leading to changes in motor, sensory, and autonomic functions [1]. Among SCI patients, males were more affected than females [2]. The common causes of SCI were similar to those reported in previous studies, except for firearm injuries (22.22%), which were more prevalent in Pakistan than in other countries [3]. This higher incidence can be attributed to aerial firing, the widespread arms culture in the region, and, notably, the terrorist activities over the past two decades, which have particularly affected the population of the province - especially in the tribal belt.

Patients suffering from SCI are prone to various secondary complications, including pain, muscle atrophy-related weakness, an increased risk of fractures, and severely restricted physical and therapeutic activities [4,5]. The cost of treating SCI places a significant burden on the healthcare system [6]. Severe and irreversible neurological deficits caused by traumatic SCI have a detrimental effect on patients' physical health and quality of life. Traditional treatments for SCI primarily aim to improve functional levels, reduce secondary complications, and enhance health-related quality of life [7]. Complications of traumatic SCI, such as fatigue, pain, pressure sores, contractures, and spasticity, impair movement and exacerbate disability. Among these, spasticity is a major challenge that negatively affects daily activities [8]. Managing spasticity remains difficult for healthcare professionals, and effective treatment is essential to improve patient outcomes. Various management techniques are used to minimize spasticity, ranging from non-invasive approaches (e.g., oral administration of antispastic medications and physiotherapy) to invasive procedures (e.g., surgical rhizotomy). The extent (diffuse vs. focal) and severity (moderate vs. severe) of spasticity determine the type and intensity of treatment required [9]. Several studies have compared different treatments for spasticity management [10]; however, there is a lack of adequate information on the subject.

In this study, we aimed to identify the most effective and affordable therapeutic management strategies for spasticity in low-income countries like Pakistan. We employed transcutaneous electrical nerve stimulation (TENS), baclofen, sustained stretching, as well as passive, assistive, and active range of motion exercises. The findings of this study will help clinicians determine the most effective treatment approach for managing traumatic SCI-related spasticity. The primary aim of this research is to evaluate the comparative efficacy of three distinct interventions: baclofen (oral), TENS, and sustained stretch, in the management of lower-limb spasticity among patients with traumatic SCI.

The specific objectives of the study are as follows: (i) to assess the therapeutic effect of oral baclofen on reducing spasticity; (ii) to evaluate the therapeutic effect of TENS application on spasticity reduction; (iii) to determine the therapeutic effect of sustained stretch on spasticity reduction; (iv) to compare the relative efficacy of these three interventions in reducing spasticity in the traumatic SCI population.

## Materials and methods

**Figure 1:**
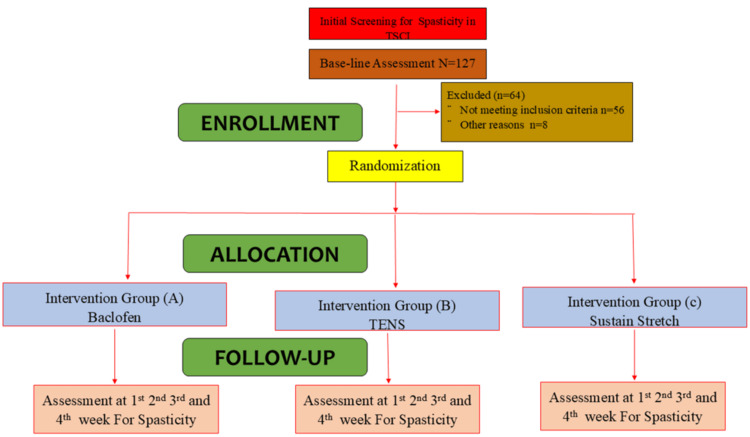
Number of participants included and excluded in each group. TENS, Transcutaneous Electrical Nerve Stimulation; TSCI, Traumatic Spinal Cord Injury

The study was conducted from March 1, 2021, to September 1, 2021. The core intervention and follow-up phase lasted four weeks, as spasticity is known to exhibit a relatively rapid and reliable response to therapeutic interventions in the short term, allowing for a timely assessment of primary outcomes. It was a single-blinded, three-arm, randomized clinical trial. Using a sealed envelope method and a non-probability convenience (consecutive) sampling technique, participants were randomly divided into three intervention groups, and each group received a separate treatment as described. Each group consisted of 21 randomly allocated participants. Figure 1 represents patient enrollment and allocation. Patients with a spasticity grade >1 and <4, according to the Modified Ashworth Score (MAS) [11], with traumatic SCI of at least three months' duration and aged between 20 and 45 years, were included. Patients were excluded if they had any chronic systemic conditions or additional major comorbidities such as hepatitis C, infections, or deep vein thrombosis (DVT); fixed contractures in the lower extremities; contraindications to baclofen, TENS, or sustained stretch (such as orthostatic hypotension, allergies, or a history of bone fracture or family history of fragility fractures); or if they were medically unstable.

Intervention protocols

Participants were divided into three groups. Group 1 (baclofen group) was treated pharmacologically with oral baclofen. Participants received 45 mg/day of baclofen, administered in three divided doses for four weeks. Group 2 (TENS group) was treated with the non-pharmacological, non-invasive TENS modality. The stimulation was applied at 100 Hz and 50 mA for 10 minutes per session, with two sessions daily during the four-week intervention period. Group 3 (sustained stretch group) was treated using the physical therapy technique of sustained stretching to the gastrocnemius muscle. The protocol involved a 20-second sustained hold, repeated for 10 repetitions per session, performed twice daily.

The outcome measure assessed the gastrocnemius muscle; however, spasticity in a flexor or extensor synergistic pattern is also very common in the lower limb after SCI. For this study, we selected one representative muscle - the gastrocnemius. The study was conducted at a specialized center for SCI: the Paraplegic Center, Peshawar. Participants also received active, assistive, and passive range of motion exercises on a daily basis. Data were gathered at the beginning of the trial (before the intervention) and then following the intervention at the first, second, third, and fourth weeks. At the end of the trial, outcomes were measured again.

The MAS was used to evaluate spasticity (0 = no increase in tone; 4 = limb rigid) [11], while the American Spinal Injury Association scoring system (ASIA scale) was used to evaluate the level of SCI [12]. The methodology exclusively utilized cost-effective and clinically practical outcome measures (MAS and ASIA scale), ensuring high relevance and applicability for practitioners in resource-limited environments.

The clinical investigator ensured that all study participants, who were traumatic SCI patients, signed a written informed consent form. The participants were well-informed about the study interventions. The informed consent was accurate, complete, easy to understand, and approved by the Institutional Review Board and Ethics Committee (approval number 082-21) of Shifa Tameer-e-Millat University, Islamabad, Pakistan. The study was also prospectively registered with the Iranian Registry of Clinical Trials (IRCT) under the identifier IRCT20210615051585N1.

IBM SPSS Statistics for Windows, Version 24 (Released 2016; IBM Corp., Armonk, NY, USA), was used to analyze the data. To examine the demographic characteristics of patients in the three therapeutic groups, we used descriptive statistics. The pre- and post-treatment values for the sustained stretch, TENS, and baclofen groups were compared using the Friedman test. A p-value of <0.05 was considered the cutoff for statistical significance, and results were presented as means. As part of each significance test, the error rate was adjusted using a post hoc correction.

## Results

There were 63 patients with traumatic SCI and lower extremity spasticity, especially in the gastrocnemius muscle, with a mean age of 30.75 ± 9.02 years. Among them, 44 (70%) were male, and 19 (30%) were female. The TENS group consisted of 16 (76%) males and 5 (24%) females; the baclofen group included 13 (62%) males and 8 (38%) females; and the sustained stretch group comprised 15 (71%) males and 6 (29%) females.

Based on the level of injury, patients were classified into C4-C9 (cervical), T2-T12 (thoracic), and L1 (lumbar) regions. Among them, five patients had tetraplegia (cervical injury), while 58 patients had paraplegia - 52 with thoracic injuries and six with lumbar injuries.

According to the ASIA scale, 53 patients had complete SCI, and 10 had incomplete SCI. In terms of ASIA scale classifications, 84.13% of patients were classified as ASIA A, 6.35% as ASIA B, 7.94% as ASIA C, and 1.59% as ASIA D (Table 1).

**Table 1:** Demographic characteristics of the patients (p < 0.05 indicates significant differences between groups). ASIA, American Spinal Cord Injury Association; TENS, Transcutaneous Electrical Nerve Stimulation; SS, Sustained Stretch

Categories		Baclofen Group		TENS Group		SS Group			Statistical Test/p-value
		n = 21	%	n = 21	%	n = 21		%	One-Way ANOVA
Age (Year)		31.10 ± 9.5		30.19 ± 9.07		30.95 ± 9.26			p = 0.943
									Chi-Square Test
Gender	Male	13	68.9	15	71.19	16	71.42		p = 0.59
	Female	8	31.1	6	23.81	5	23.81		
Cause of Injury	Fall From Height	7	33.33	7	33.33	9	42.85		p = 0.203
	Firearm Injury	9	42.85	2	9.52	3	14.28		
	Road Traffic Accident	2	9.52	6	28.57	5	23.80		
	Hit by Falling Object	1	4.76	3	14.28	1	4.76		
	Act of Violence	0	0	2	9.52	1	4.76		
	Spinal Cord Injury Post-opp	0	0	0	0	1	4.76		
	Earthquake Victims	2	9.52	0	0	1	4.76		
	Coal Mine Accident	0	0	1	4.76	0	0		
Level of Injury	Thoracic	18	85.71	19	90.47	15	71.42		p = 0.367
	Cervical	2	9.52	0	0	3	14.28		
	Lumbar	1	4.76	2	9.52	3	14.28		
ASIA Score	A	19	90.48	18	85.71	16	76.19		p = 0.588
	B	0	0	2	9.52	2	9.52		
	C	2	9.52	1	4.76	2	9.52		
	D	0	0	0	0	1	4.76		

Effect of sustained stretch on spasticity

Spasticity of the gastrocnemius muscle was assessed using the MAS at baseline; the first week; and the second, third, and fourth weeks post-treatment. A significant decrease (p < 0.001) in spasticity was observed at week 4 compared to baseline. The baseline MAS mean rank was 4.17. After the first week, the MAS decreased to 3.07, with further significant reductions in the second, third, and fourth weeks to 2.57, 2.90, and 2.29, respectively.

**Figure 2:**
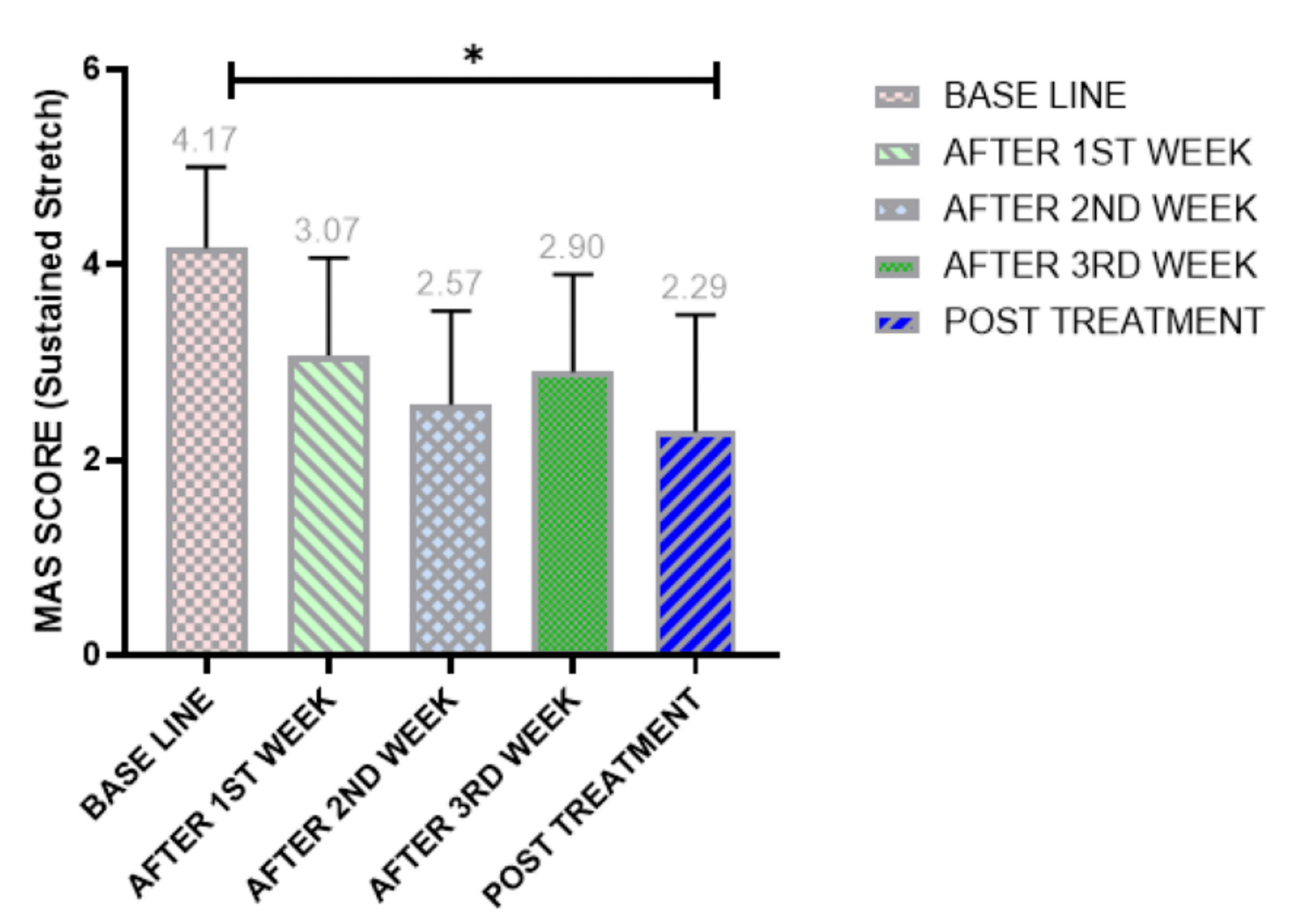
Score assessment of the MAS showing the effect of sustained stretch on all patients at each time point. Friedman test: *p < 0.05 indicates a significant difference. MAS, Modified Ashworth Scale

Within-group analysis using Friedman's test revealed statistically significant differences (p < 0.001). Friedman's two-way analysis of variance by rank confirmed significant differences between baseline and all subsequent weeks (p < 0.001). Pairwise comparisons showed significant variations at the fourth week post-intervention compared to baseline, while non-significant differences were observed at the first, second, and third weeks. Post hoc analysis using the Bonferroni test confirmed significant differences between baseline and post-treatment periods (Figure 2).

Electrophysiological effect of spasticity

MAS before and four weeks after receiving TENS demonstrated a decrease in spasticity of the gastrocnemius muscle across all weeks. The baseline MAS mean rank was 3.83, reducing to 3.40 after the first week, with further significant reductions in the second, third, and fourth weeks - to 2.83, 2.88, and 2.05, respectively.

**Figure 3:**
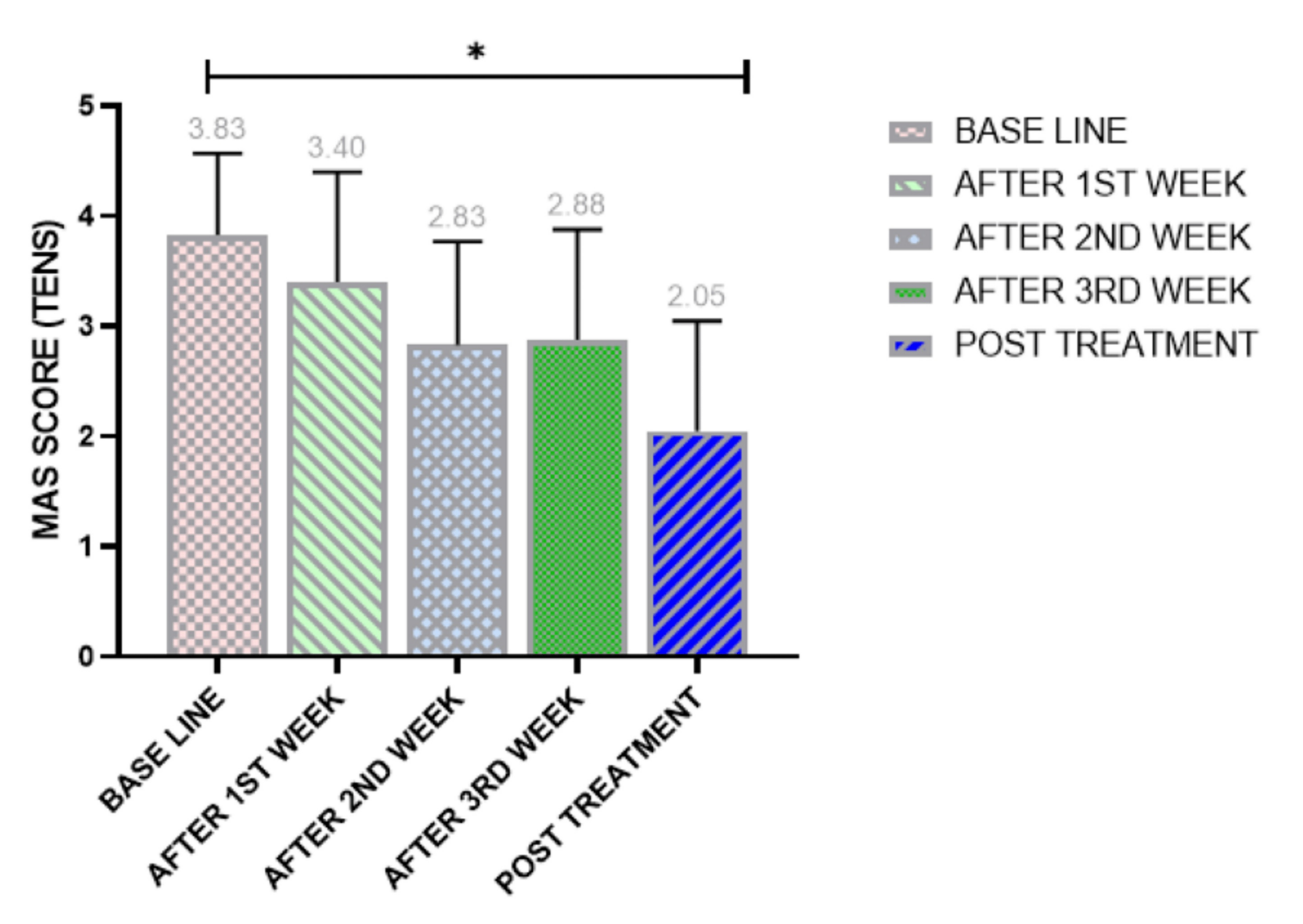
Score assessment of the MAS for the TENS effect of all patients at each time point. Friedman test: *p < 0.05 indicates a significant difference. TENS, Transcutaneous Electrical Nerve Stimulation; MAS, Modified Ashworth Scale

Within-group analysis using Friedman's test showed a statistically significant difference (p < 0.001) at baseline and across all weeks. Friedman's two-way analysis of variance by rank confirmed a statistically significant difference between baseline and all post-intervention weeks (p < 0.001) (Figure 3).

Pharmacological effect of spasticity

**Figure 4:**
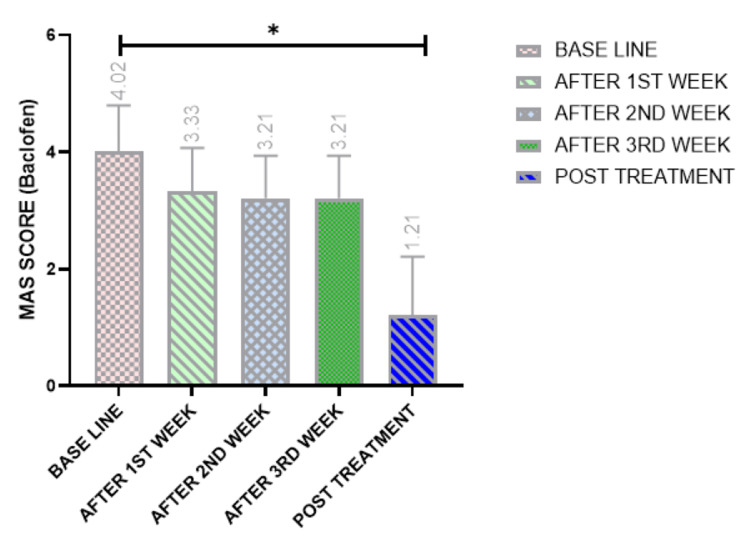
Score assessment of the MAS showing the effect of baclofen on all patients at each time point. Friedman test: *p < 0.05 indicates a significant difference. MAS, Modified Ashworth Scale

Changes in spasticity before and four weeks after receiving baclofen are shown in Figure 4. A decrease in MAS was observed across all weeks. The baseline MAS mean rank was 4.02, reducing to 3.33 after the first week (p < 0.001). Significant decreases were also observed in the second, third, and fourth weeks, with MAS scores of 3.21, 3.21, and 1.21, respectively.

Within-group analysis using Friedman’s test showed a statistically significant difference (p < 0.001). Friedman’s two-way analysis of variance by rank also revealed significant differences between baseline and all post-intervention weeks (p < 0.001). Pairwise comparisons demonstrated significant differences at the fourth week post-intervention compared to baseline, as well as the first, second, and third weeks. However, no significant differences were found between the treatment weeks themselves (i.e., first vs. second, second vs. third, and third vs. first). Post hoc analysis further confirmed significant differences at the fourth week post-intervention, compared to baseline and all previous weeks.

Comparison between sustained stretch, TENS, and baclofen

The Kruskal-Wallis test was used to compare the sustained stretch, TENS, and baclofen groups at baseline and after the first, second, third, and fourth weeks of intervention. Results indicated no significant differences in MAS scores for the gastrocnemius muscle in the lower limb at baseline among all intervention groups (p = 0.393), and no significant differences after the first, second, and third weeks (p-values of 0.983, 0.576, and 0.614, respectively). However, a significant difference was observed at the fourth week post-intervention (p = 0.022) (Table 2).

**Table 2:** Comparison between sustained stretches, TENS, and baclofen groups. *Significant between groups (p < 0.05). TENS, Transcutaneous Electrical Nerve Stimulation

Duration	Intervention Groups	Kruskal-Wallis Test
Baseline	TENS	p = 0.393
	Sustained Stretch	
	Baclofen	
First Week	TENS	p = 0.983
	Sustained Stretch	
	Baclofen	
Second Week	TENS	p = 0.576
	Sustained Stretch	
	Baclofen	
Third Week	TENS	p = 0.614
	Sustained Stretch	
	Baclofen	
Fourth Week	TENS	p = 0.022^*^
	Sustained Stretch	
	Baclofen	

## Discussion

Several studies have explored various approaches for managing spasticity in SCI patients. The most effective strategy for managing spasticity is to understand the risks and benefits of different therapeutic options. The average age of participants in the baclofen group was 31.10 ± 9.5 years; in the TENS group, 30.19 ± 9.07 years; and in the sustained stretch group, 30.95 ± 9.26 years, with a male predominance of 70%. Shaygannejad et al. conducted a comparative study on TENS and baclofen, with 26 individuals in each group. The mean age of participants in their study was 39.5 ± 9.3 years and 38.9 ± 7.8 years, respectively [13].

The common causes of SCI in our study were consistent with previous research, except for firearm injuries, which were higher in Pakistan (22.22%). Darain et al. conducted an epidemiological study and reported a firearm injury rate of 21.1% [3].

According to Holtz et al., antispasmodic medications were frequently used by practitioners for managing spasticity. Their study showed that 32% of hospitalized patients used medication for spasticity control [14]. In comparison, in our study, baclofen was administered to 33.33% of participants. Baclofen has been found to reduce lower limb spasticity more effectively than upper limb spasticity, and in our study, we also assessed MAS scores for spasticity in the gastrocnemius muscle of the lower limb [15]. A study conducted by Cragg et al. on a large number of participants taking therapeutic doses of baclofen found it to be a safe drug for spasticity treatment; however, our study also did not find any adverse effects [16].

Previous studies have indicated that higher doses of baclofen are required to manage symptoms effectively [17]. A dose of up to 80-90 mg/day is typically used for moderate to severe spasticity, while doses up to 200 mg/day are tolerated in SCI patients [18]. In our study, we administered 45 mg/day of baclofen for four weeks, which showed significant effects in managing spasticity in traumatic SCI patients. Our findings confirmed that baclofen reduced spasticity. A 44% reduction was observed in the baclofen group, aligning with previous research. Romito et al. also found that baclofen had a positive impact on spasticity, though it was associated with certain side effects [19].

Previously reported side effects of baclofen include muscle hypotonia, nausea, and vertigo, which are generally mild to moderate in severity and reversible [20]. Baclofen overdose and toxicity are more likely when exceeding the recommended dosage for spasticity. The most severe adverse effects of baclofen toxicity include hypotonia (flaccid paralysis), while, conversely, withdrawal from baclofen can lead to hyperreflexia and worsening spasticity [19].

To understand the role of TENS in spasticity therapy, its effects over short and long durations must be examined. Most studies have focused on its short-term effects [17]. In our study, TENS was applied to the gastrocnemius muscle in the lower limb for 10 minutes, twice daily. In contrast, in a study by Hsieh et al., TENS was applied once daily for the same duration, and their results also showed a reduction in spasticity [21].

Another study applied TENS for 30 minutes at a frequency of 50 Hz, improving several measures of lower extremity spasticity for up to two hours post-intervention in 12 individuals with chronic SCI [22]. The MAS was used as a reliable and effective tool to assess spasticity [23]. Previous studies applied low-amplitude stimulation to target muscles without inducing muscle contractions, using frequencies between 50 and 100 Hz [24,25]. However, these studies applied TENS for a short duration, whereas our study extended TENS application to four weeks, resulting in a significant reduction in spasticity in the gastrocnemius muscle of the lower extremity. The TENS intervention group showed a notable reduction in spasticity within the group.

A study conducted on 44 traumatic SCI patients reported a reduction in spasticity. In that study, 35 spastic patients who did not receive electrical stimulation served as a control group. The findings demonstrated that electrical stimulation decreased spasticity over a long period and improved both active and passive range of motion [26,27]. Another study comparing different electrical stimulation techniques for treating triceps surae spasticity in SCI included 10 patients with complete SCI who were unable to voluntarily contract their triceps surae muscle. When compared to a placebo strategy, stimulation of the agonist muscle resulted in a significant decrease in MAS (p < 0.001). The study concluded that stimulating the triceps surae muscle reduced the MAS for that specific muscle [6], supporting the findings of our study, which also demonstrated decreased MAS among participants.

Sustained stretching plays a crucial role in preventing contractures and reducing motor neuron excitability (spasticity) over time [28]. In our study, significant improvements in left and right ankle dorsiflexion range of motion were observed, which is a function of the gastrocnemius muscle, in the intervention group following sustained muscle stretching and passive exercises [29]. Dorin-Alexandru et al. conducted a study in which they applied 10-15 seconds of sustained stretch to the targeted muscle. The MAS demonstrated statistically significant improvements in spasticity, with each muscle receiving three sets of five repetitions. Additionally, improvements were noted in the 10 m walk test, knee and ankle joint range of motion, ambulatory speed, and walking ability [30]. Another study suggested that a minimum of 20 seconds of sustained stretching is required for effectiveness. Eligible stretch interventions included sustained passive stretching, positioning, splinting, and serial casting [26]. Our sustained stretching intervention group also showed a significant reduction in spasticity. Regular stretching exercises during physiotherapy sessions for SCI patients were found to have short-term beneficial effects, reducing spasticity and enhancing walking ability [26,30]. Our study followed the intervention duration of previous studies, applying a 20-second sustained stretch. The results demonstrated a significant reduction in spasticity within the group. While baclofen showed a rapid effect after administration, it was associated with side effects, as reported in previous studies. In contrast, the superiority of the sustained stretch intervention is clarified by its combination of strong, comparable long-term efficacy and its superior safety profile (no observed adverse effects), making it the most practical and sustainable choice, especially in low-resource settings.

## Conclusions

This randomized clinical trial found that oral baclofen, TENS, and sustained stretch all significantly reduce spasticity in patients with traumatic SCI. Among them, sustained stretch emerged as the most suitable, showing earlier and consistent improvements with no side effects, making it safe, practical, and cost-effective for low-resource settings. Baclofen demonstrated rapid effectiveness but carries risks of drowsiness and weakness. TENS was effective but produced slower improvements and occasional skin irritation. Overall, sustained stretch is recommended as the primary non-pharmacological and long-term management strategy for spasticity in individuals with traumatic SCI.
